# Neighborhood disadvantage and adolescent stress reactivity

**DOI:** 10.3389/fnhum.2012.00277

**Published:** 2012-10-12

**Authors:** Daniel A. Hackman, Laura M. Betancourt, Nancy L. Brodsky, Hallam Hurt, Martha J. Farah

**Affiliations:** ^1^Department of Psychology, Center for Neuroscience and Society, Center for Cognitive Neuroscience, University of PennsylvaniaPhiladelphia, PA, USA; ^2^Division of Neonatology, Department of Pediatrics, The Children's Hospital of PhiladelphiaPhiladelphia, PA, USA

**Keywords:** socioeconomic status, neighborhood disadvantage, parental education, stress reactivity, cortisol, HPA axis

## Abstract

Lower socioeconomic status (SES) is associated with higher levels of life stress, which in turn affect stress physiology. SES is related to basal cortisol and diurnal change, but it is not clear if SES is associated with cortisol reactivity to stress. To address this question, we examined the relationship between two indices of SES, parental education and concentrated neighborhood disadvantage, and the cortisol reactivity of African–American adolescents to a modified version of the Trier Social Stress Test (TSST). We found that concentrated disadvantage was associated with cortisol reactivity and this relationship was moderated by gender, such that higher concentrated disadvantage predicted higher cortisol reactivity and steeper recovery in boys but not in girls. Parental education, alone or as moderated by gender, did not predict reactivity or recovery, while neither education nor concentrated disadvantage predicted estimates of baseline cortisol. This finding is consistent with animal literature showing differential vulnerability, by gender, to the effects of adverse early experience on stress regulation and the differential effects of neighborhood disadvantage in adolescent males and females. This suggests that the mechanisms underlying SES differences in brain development and particularly reactivity to environmental stressors may vary across genders.

Socioeconomic status (SES), particularly childhood SES, is consistently associated with disparities in disease morbidity and mortality as well as cognitive performance, academic achievement, depression, anxiety, and behavior problems (Adler et al., 1994; Brooks-Gunn and Duncan, 1997; McLoyd, 1998; Bradley and Corwyn, 2002; Chen et al., 2002; Costello et al., 2003; Wadsworth and Achenbach, 2005). Children and adolescents of lower SES report higher levels stress and are routinely exposed to increased family turmoil and more dangerous, crowded and polluted neighborhoods (Brady and Matthews, 2002; Evans, 2004; Goodman et al., 2005). Such chronic stress generates adaptations in the underlying psychological and biological systems that regulate responses to environmental stressors, leading to increased vulnerability to disease and disorder (Adler et al., 1994; Gallo and Matthews, 2003; Shonkoff et al., 2009; Hackman et al., 2010; McEwen and Gianaros, 2010; Miller et al., 2011).

One stress response system, the hypothalamic-pituitary-adrenal (HPA) axis, ultimately produces cortisol in response to physical or psychological threats to well-being. As the function of this system is associated with social factors and is implicated in health and well-being (Gunnar and Quevedo, 2007), it is a plausible mechanism underlying the emergence of disparities. Multiple indices of SES, typically income, education, occupation, or some combination thereof, have been shown to predict differences in baseline measures of cortisol, the diurnal slope of cortisol, total cortisol exposure throughout the day, and the cortisol awakening response in adults (Kristenson et al., 2001; Steptoe et al., 2003; Cohen et al., 2006a,b; Gustafsson et al., 2010, 2011; Hajat et al., 2010; Dowd et al., 2011). In children, family SES has also been shown to predict total overnight cortisol concentration, diurnal slopes, and baseline measures of cortisol in children as well, though the direction of findings has not always been consistent and it is unclear if these relationships extend into adolescence (Lupien et al., 2000, 2001; Evans and English, 2002; Chen and Paterson, 2006; Gustafsson et al., 2006; Evans and Kim, 2007; Chen et al., 2010; Zalewski et al., 2012).

Although response to acute threat is the primary function of the stress system, few studies have investigated the relationship between SES and cortisol reactivity to stress. In adults, education has been both positively (Neupert et al., 2006) and negatively (Fiocco et al., 2007) related to cortisol reactivity, while multiple studies have found no differences in reactivity by SES (Kapuku et al., 2002; Steptoe et al., 2005; Kraft and Luecken, 2009). Even when observed, the causal direction of such effects is difficult to ascertain, as it stands to reason that those who can more successfully manage and respond to performance-based, socially evaluative stressors are more likely to exhibit greater educational achievement in adulthood. In children and adolescents, however, such individual-level social selection is unlikely. In 5-year olds, Blair et al. (2005) found that lower income-to-needs, in a low-SES sample, predicted increased cortisol reactivity. A similar effect was found in children 9.5 years old from a sample with a broader income range (Gump et al., 2009). However, a quasi-experimental intervention designed to relieve extreme poverty through cash payments along with health and educational programs had no effect on cortisol reactivity in children ages 2–6 (Fernald and Gunnar, 2009). In addition, few such studies employ reactivity protocols that enhanced the aspects of social evaluative threat and uncontrollability involved in eliciting a stress response with a standardized protocol (Dickerson and Kemeny, 2004; Gunnar et al., 2009).

To date, studies of cortisol reactivity and SES have also largely omitted investigation of neighborhood components of disadvantage. Neighborhood differences represent a separate and meaningful aspect of SES that is distinct from family based measures of income or education (Krieger et al., 1997; Duncan and Magnuson, 2003; Braveman et al., 2005). Neighborhood disadvantage has been implicated as a predictor of disease morbidity and mortality (Kawachi and Berkman, 2003), lower scores on tasks of verbal ability (Sampson et al., 2008), and reduced serotonergic responsivity to challenge (Manuck et al., 2005). Moreover, experimental data from the Moving to Opportunities (MTO) study have primarily found that moving to more affluent neighborhoods resulted in considerable changes in socioemotional functioning for both adolescents and adults (Sanbonmatsu et al., 2006; Kling et al., 2007). With respect to HPA axis function, lower neighborhood SES predicts lower morning cortisol in adults (Dulin-Keita et al., 2012) and, independently of family SES, lower afternoon cortisol in adolescents (Chen and Paterson, 2006). Only one study has investigated its relationship to reactivity: Kapuku et al. (2002) found no relationship between neighborhood SES and cortisol reactivity in a small sample of 16- to 25-year old males.

Experimental and quasi-experimental research with animals and humans suggests that the relationship between cortisol reactivity and SES, particularly neighborhood disadvantage, may be moderated by gender. Research with animal models indicates that the effect of stressors on HPA axis function and on the development of stress-related brain regions is moderated by gender (McCormick and Mathews, 2007; Weinstock, 2007; Lin et al., 2009). The MTO intervention found that the effects of moving to more affluent neighborhoods varied by gender, with positive effects on mental health and problem behaviors for girls and negative effects for boys (Kling et al., 2007). Moreover, non-experimental changes in neighborhood disadvantage predict changes in boys, internalizing and externalizing problems, with far smaller associations in girls (Leventhal and Brooks-Gunn, 2011). Consequently, the limited or mixed findings concerning SES and cortisol reactivity in the literature may be due to omission of neighborhood SES as well as incomplete consideration of the moderating effects of gender.

The current analysis was designed to address these limitations by examining the relationship between cortisol reactivity and both neighborhood disadvantage and parental education, as moderated by gender, in African–American adolescents. Adolescent participants were exposed to a mild social stressor, a modified version of the Trier Social Stress Test (TSST) (Kirschbaum et al., 1993; Childs et al., 2006; von Dawans et al., 2011). Consequently, this study is uniquely positioned to determine if parental education and neighborhood disadvantage, as moderated by gender, are associated with cortisol reactivity to stress.

## Materials and methods

### Participants

Participants were 79 African–American adolescents drawn from the control group of a larger longitudinal study of prenatal cocaine exposure (*n* = 55) and a cohort of adolescents, also not exposed to cocaine prenatally, recruited for an earlier study of SES and neurocognitive development (*n* = 24) (Hurt et al., 1995; Farah et al., 2006). Detailed descriptions of participant characteristics were reported previously (Farah et al., 2006; Hurt et al., 2009). One participant did not sleep the night before the stressor protocol and was thus excluded from the analysis, while another was excluded because cortisol values were greater than 3 SD above the mean of the other participants on seven of nine samples. These two samples were combined, and thus analyses included a total of 77 participants (37 female, 48.1%) between the ages of 13 and 18 (*M* = 16.4, SD = 1.2). Consent was obtained from participants aged 18 and older. For participants younger than age 18 both parental or guardian consent and child assent were obtained. The project was approved by the Institutional Review Boards of the University of Pennsylvania and The Children's Hospital of Philadelphia.

### Indicators of socioeconomic status

We employed a measure of concentrated neighborhood disadvantage based on the 2000 United States (U.S.) census tract for the participant's home address when the stress reactivity protocol was completed (Sampson et al., 1997, 2008). The 77 participants lived in 59 census tracts. Six measures were employed: percentage of individuals below the poverty line, unemployed, and receiving public assistance, as well as the density of African–Americans, children under the age of 18, and female-headed households. Principal components analysis confirmed a single factor of concentrated disadvantage which accounted for 71.4% of the total variance in the six variables. Factor loadings are presented in Table 1, which were used to create a continuous, regression-weighted factor score for concentrated disadvantage. Distribution of this factor was within limits for normality.

**Table 1:** **Neighborhood characteristics and concentrated disadvantage factor loading (*n* = 77)**.

Neighborhood measure^*^	***M* (SD)**	**Range (min–max)**	**Inter-quartile range**	Above US average (%)^**^	**Factor loading**
Below poverty	23.0 (14.7)	0.0–59.0	12.1–31.7	71.4	0.90
Unemployed	12.5 (6.7)	0.0–27.9	8.0–16.5	85.7	0.92
Public assistance	10.3 (7.2)	0.0–28.2	4.7–14.6	81.8	0.94
African–American	63.8 (36.9)	1.3–97.4	26.0–95.4	87.0	0.60
Under 18	28.3 (5.1)	14.4–43.7	25.0–31.6	71.4	0.71
Female-only household	28.0 (10.4)	4.9–43.4	19.2–36.3	87.0	0.94

* Expressed as the percentage of families in the census tract.

** Percentage of census tracts above US Average.

Parental education was scored as the following for the participant's primary caregiver: raw number of years for those who did not complete high school (score up to 11); 12 for those who completed high school or the General Educational Development (GED) test; 14 for those who completed an Associates degree, some college, or vocational school; 16 for those who completed a Bachelor's degree; 18 for those who competed a Master's degree or some graduate work; 19 for those who completed a Law degree; and 21 for those who completed a Doctoral-level degree (i.e., EdD, MD, PhD, etc.).

### Stressor protocol and procedure

Participants completed the Trier Social Stress Test for Groups (TSST-G) (von Dawans et al., 2011) a modified version of the TSST that induces a moderate level of stress (Kirschbaum et al., 1993; Dickerson and Kemeny, 2004). In order to increase social evaluation (Dickerson and Kemeny, 2004) participants underwent the protocol in groups of 2(*n* = 27, 35.1%) or 3(*n* = 50, 64.9%) (Childs et al., 2006; von Dawans et al., 2011), matched for gender.

Participants were contacted the evening before their session and instructed to refrain from consuming a major meal 60 min before their session, drinking milk or eating other dairy products 30 min before the appointment, eating acidic or high sugar foods 20 min before the appointment, brushing teeth within 1 h before their session, and consuming alcohol in the 12 h prior to the session (Salimetrics, LLC, State College, PA). All sessions began between 11:30 am and 3:30 pm to control for the diurnal pattern of cortisol, with 84.4% of appointments at 1:00 pm or 1:30 pm.

**Figure 1:**
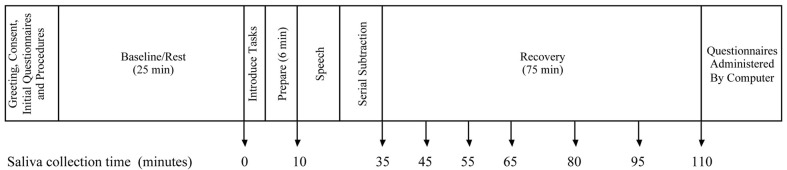
**Timeline of procedures**.

Figure 1 outlines the protocol for the stress induction procedure. Upon arrival, participants were greeted by the experimenter and directed to sit in a semi-private room where they were able to interact with an experimenter but not with other participants. After completion of the consent process, experimenters conducted a short interview to assess participant compliance with pre-appointment instructions and to survey use of prescribed and non-prescribed medications. To establish a baseline prior to stress induction, participants watched a video with minimally arousing content for 25 min. Participants then performed stressor tasks in a testing room with other group members and an unfamiliar experimenter dressed in a lab coat acting as a judge and directing the testing room activities. To enhance the social evaluative component of the stressor (Dickerson and Kemeny, 2004) participants were told their performance was being videotaped and scored. They were given 6 min to prepare a 3-min speech promoting their candidacy for a summer job, and they each gave their speech facing the video camera, the other participants, and the judge. Subsequently they were instructed to perform serial subtraction by eights, aloud, for 3 min, in front of the same audience. Individuals were given unique four-digit numbers as starting points. If subtraction mistakes were made they were told to re-start from the beginning. Participants were randomly assigned to perform each task first, second, or third. After all participants completed the stressor task they returned to the semi-private room for a 75 min recovery period during which they continued watching the video shown during baseline.

### Measures of the stress response

Salivary cortisol was the primary outcome of interest, and saliva samples were collected at nine different times: at baseline, after speech preparation, at the end of the stressor tasks, and 10, 20, 30, 45, 60, and 75 min after the stressor (see Figure 1). Saliva samples were collected using the passive drool technique to avoid the potential interference introduced when using oral stimulants to assist in generating saliva (Schwartz et al., 1998; Talge et al., 2005). Samples were frozen immediately at −70°C and transported on dry ice to Salimetrics, LLC (State College, PA) for analysis using enzyme immunoassay techniques. Assays were conducted in duplicate and average cortisol concentrations were used. The test uses 25 μl of saliva per determination has a lower limit of sensitivity of 0.003 μg/dl and standard curve range from 0.012 to 3.0 μg/dl. Intra- and inter-assay coefficients of variation were 3.5 and 5.1% respectively. Due to skewed distributions, the natural log of the average cortisol concentration was the primary dependent measure.

Subjective responses to the protocol were collected during the procedure as well as retrospectively. Participants rated their anxiety level concurrent with the collection of saliva samples using a seven-point Likert-type scale (1 = very calm and relaxed; 3 = feeling pretty calm and relaxed; 5 = a little bit nervous, but not too bad; 7 = very nervous or stressed). After completion of the stressor, at the start of the recovery period, participants were asked how stressful and challenging they found the speech and math tasks, with response choices structured along a seven-point Likert type scale (1 = not at all challenging or stressful, 7 = extremely challenging or stressful).

### Covariates

Multiple potential confounds were measured and included in analyses in order to rule out effects due to methodological factors and additional participant behaviors that may affect stress responses. Participants' use of prescription and over-the-counter medications, as well as oral contraceptives, was assessed (Kirschbaum et al., 1999; Hibel et al., 2006). Of all classes of medications, only oral contraceptives (*n* = 6), non-steroidal asthma medication (*n* = 5), ibuprofen (*n* = 5), and acetaminophen (*n* = 8) were used by more than three participants and thus included in analyses. Five participants currently smoked cigarettes (6.5%) (Rohleder and Kirschbaum, 2006). Mean hours of sleep the night before was 7.8 (SD = 2.1) while the average time participants had been awake at the beginning of the protocol was 5.7 h (SD = 2.0) (Leproult et al., 1997; Spiegel et al., 1999).

### Data analysis

Piecewise hierarchical linear modeling (HLM) was the primary analysis strategy^1^. Piecewise HLM is a strategy based on longitudinal growth modeling that allows distinct modeling of the different phases of change over time, permits the separate modeling of reactivity and recovery phases following administration of a stressor, and offers advantages over the use of ANOVA or change scores (Llabre et al., 2001; Hruschka et al., 2005; Bernard and Dozier, 2010). A Level-1 model was estimated that represents the individual change in the natural log of salivary cortisol across the protocol and included both fixed components and random components (intercept and slopes) that were permitted to vary across individuals. Time was recoded into two separate components, to create a two-piece linear model. The first component represents time linearly from baseline through the measures of cortisol taken 10 min after the completion of the stressor (minute 45), capturing the episode of reactivity to the stressor. Saliva collection times (see Figure 1) for the reactivity episode were thus coded, in minutes, as 0, 10, 35, 45, 45, 45, 45, 45, and 45. The second linear component represents the episode of recovery from the stressor, the time from 10 min after the completion of the stressor through the end of the protocol. Saliva collection times for the recovery episode were thus coded as 0, 0, 0, 0, 10, 20, 35, 50, and 65. This results in the following Level-1 model:

(1) $$\ln{\left(\text{cort}\right)}_{ti}=\;\pi_{0i}+\pi_{1i}{\left(\text{Reactivity}\;\text{episode}\right)}_{ti}+\pi_{2i}{\left(\text{Recovery}\;\text{episode}\right)}_{ti}+\varepsilon_{ti}$$

In this model, the natural log of salivary cortisol is predicted by π_0*i*_, the intercept, π_1*i*_, the linear rate of change during reactivity and π_2*i*_, the linear rate of change during recovery. Given the coding of time employed for reactivity and recovery, the intercept, π_0*i*_, is an estimate of the natural log of cortisol concentration at baseline before administration of the stressor. Level-2 equations were also estimated in which the variance in the intercept and slope parameters at Level-1 were predicted by parental education and neighborhood disadvantage, as continuous variables, and their interaction with gender. Analyses were conducted in HLM6 (Raudenbush et al., 2004) using full maximum likelihood estimation. All variables included in analyses were grand-mean centered. Descriptive data were analyzed using SPSS 20.0 (IBM: New York, NY).

First an unconditional linear piecewise growth model was created, and then separate models were created for neighborhood disadvantage and parental education as independent variables at Level-2. Each model included gender and the interaction of either education or disadvantage with gender. For example, for neighborhood disadvantage, the basic Level-2 equations were as follows:

(2) $$\pi_{0i}=\;\beta_{00}+\beta_{01}{\left(\text{Disadvantage}\right)}_{i}+\beta_{02}{\left(\text{Gender}\right)}_{i}+\beta_{03}{\left(\text{Disadvantage}\times \text{Gender}\right)}_{i}+\zeta_{0i}$$

(3) $$\pi_{1i}=\;\beta_{10}+\beta_{11}{\left(\text{Disadvantage}\right)}_{i}+\beta_{12}{\left(\text{Gender}\right)}_{i}+\beta_{13}{\left(\text{Disadvantage}\times \text{Gender}\right)}_{i}+\zeta_{1i}$$

(4) $$\pi_{2i}=\;\beta_{20}+\beta_{21}{\left(\text{Disadvantage}\right)}_{i}+\beta_{22}{\left(\text{Gender}\right)}_{i}+\beta_{23}{\left(\text{Disadvantage}\times \text{Gender}\right)}_{i}+\zeta_{2i}$$

We then included potential control variables individually and noted each variable which was significant at a level of *p* < 0.10 and for which the fit of the prediction model was improved (Singer and Willett, 2003). These variables were gender, age, group size, current cigarette smoking, hours of sleep the night before, hours since awakening, and the use of oral contraceptives, non-steroidal asthma medication, ibuprofen, and acetaminophen. Next, we created a basic prediction model including all variables identified in the previous step and then removed non-significant (*p* > 0.05) control variables sequentially starting with the highest *p*-value, until only significant control variables remained. Significant interaction effects were then examined by re-centering gender in two models, with males and females coded as 0 and 1 in one model, and 1 and 0 in the second model. Consequently, the slope coefficient of concentrated disadvantage in each model is that for the gender coded as 0; by re-centering in this manner, estimates of the effect of concentrated disadvantage for each gender are obtained. Finally, we examined if findings from this complete prediction model were explained by differences in subjective appraisals of and responses to the stressor.

## Results

### Sample characteristics: socioeconomic status

Characteristics of the 59 unique census tracts participants lived in are delineated in Table 1. Across these 59 tracts, on average, 23.0% (SD = 14.7) of individuals were below the poverty line, with percentages below the poverty line ranging from 0 to 59.0%. Neighborhood unemployment averaged 12.5% (SD = 6.7), while 10.3% (SD = 7.2) of neighborhood residents were receiving public assistance. These levels are above the United States average, and range from neighborhoods with the near absence of disadvantage to neighborhoods with highly concentrated disadvantage. Descriptive statistics for parental education are presented in Table 2. Average parental education for families was 13.5 (SD = 3.0), and ranged from 6 to 21 years. Eight participants (10.4%) had a primary caregiver with less than a high school education while 40 participants (51.9%) had a primary caregiver with a high school education. Parental education and concentrated disadvantage were correlated (*r* = −0.55, *p* < 0.001), such that lower parental education was related to higher levels of neighborhood disadvantage.

**Table 2:** **Parental education, years (*n* = 77)**.

	**Parental education**
**SUMMARY MEASURES**	
Mean (SD)	13.5 (3.0)
Range (min–max)	6–21
Interquartile range	12–15
**DISTRIBUTION [NUMBER (%)]**	
Less than 12 years	8 (10.4)
12 years	40 (51.9)
Associates degree or some college	10 (13.0)
Bachelor's degree	7 (9.1)
Graduate degree	12 (15.6)

### Subjective appraisal of the stressor

Peak anxiety ratings during the stressor were in the moderate range (*M* = 4.5, SD = 1.5), while the average change in rating from baseline to peak was 2.4 (SD = 1.7) on the seven-point Likert scale. After stressor administration, both the math (*M* = 4.4, SD = 1.8) and speech (*M* = 4.5, SD = 1.7) tasks were rated as moderately challenging on a seven-point scale, while the overall protocol was rated as moderately stressful (*M* = 4.0, SD = 1.9).

### Predicting baseline cortisol, reactivity, and recovery: interaction between gender and concentrated disadvantage

Seventy-four participants (96.1%) had complete data for salivary cortisol at Level 1, while three participants each were missing one data point, for a total of 690 Level 1 observations. The unconditional piecewise growth model for cortisol level over time across the protocol yielded a non-significant, positive fixed effect for the reactivity episode (*B* = 0.007, *p* = 0.64) and a significant fixed effect for the recovery episode, in the negative direction (*B* = −0.007, *p* < 0.001). However, the random effects for the intercept (σ^2^_0_ = 0.22, *p* < 0.001) estimating baseline cortisol before the stressor, the slope of the reactivity episode (σ^2^_1_ = 0.0015, *p* < 0.001), and the slope of the recovery episode (σ^2^_2_ = 0.0004, *p* < 0.001) were all significant. This indicates that, on average, the stress manipulation did not generate an increase in cortisol above baseline. However, there are significant individual differences in intercept as well as the slope during reactivity and recovery, such that some participants exhibited increases in cortisol while others did not, and consequently there is sufficient variance to predict systematically with indicators of SES and gender.

As illustrated in Table 3, Model A, the main effect of concentrated disadvantage on reactivity was borderline significant (*B*= 0.003, *p* = 0.052, *r*_effect_ = 0.23). However, the interaction between gender and concentrated disadvantage was significant for both reactivity (*B* = −0.006, *p* = 0.03, *r*_effect_ = 0.25) and recovery (*B* = 0.004, *p* = 0.009, *r*_effect_ = 0.30). Neither concentrated disadvantage (*B* = −0.07, *p* = 0.19, *r*_effect_ = 0.15) nor its interaction with gender (*B* = −0.03, *p* = 0.81, *r*_effect_ = 0.03) predicted the intercept, or baseline prior to the stressor. This effect was specific to the neighborhood measure of SES; as described in Table 3, Model B, there were no significant effects on the intercept, reactivity, or recovery of parental education (all *p* ≥ 0.12) or its interaction with gender (all *p* ≥ 0.10)^2^.

**Table 3:** **Linear piecewise model of salivary cortisol**.

**Parameter**	**Model A: Concentrated Disadvantage**		**Model B: Parental Education**	
	***B***	***r*_effect_**	***B***	***r*_effect_**
**FIXED EFFECTS**				
Initial status, π_0*i*_				
Intercept	−2.00^***^	0.98	−1.99^***^	0.97
Concentrated disadvantage	−0.07	0.15		
Parental education			0.02	0.13
Gender	0.27^*^	0.28	0.29^**^	0.30
Disadvantage × Gender	−0.03	0.03		
Education × Gender			0.03	0.09
Sleep	−0.05^*^	0.26	−0.05^*^	0.25
Episode 1, reactivity, π_1*i*_				
Intercept	0.001	0.07	0.001	0.10
Concentrated disadvantage	0.003^∧^	0.23		
Parental education			0.000	0.08
Gender	−0.006^*^	0.27	−0.0007^*^	0.26
Disadvantage × Gender	−0.006^*^	0.25		
Education × Gender			0.002	0.18
Asthma med (non-steroidal)	0.011^*^	0.28	0.01^**^	0.31
Episode 2, recovery, π_2*i*_				
Intercept	−0.007^***^	0.74	−0.007^***^	0.73
Concentrated disadvantage	−0.001	0.18		
Parental education			0.000	0.18
Gender	−0.001	0.09	−0.001	0.06
Disadvantage × Gender	0.004^**^	0.30		
Education × Gender			−0.001^∧^	0.19
	**Estimate**	**SE**	**Estimate**	**SE**
**RANDOM EFFECTS**				
Level 1				
Within-person, σ^2^_ε_	0.042	0.0027	0.042	0.0027
Level 2				
Initial status, σ^2^_0_	0.194	0.035	0.194	0.036
Episode 1, reactivity, σ^2^_1_	0.0001	0.00002	0.0001	0.0000
Episode 2, recovery, σ^2^_2_	0.00003	0.00001	0.0000	0.0000
Covariance, σ^1^_0_	−0.0017	0.0007	0.0019	0.0007
Covariance, σ^2^_0_	−0.0007	0.0004	−0.0006	0.0004
Covariance, σ^2^_1_	−0.00002	0.00001	0.0000	0.0000
*R*^2^_ε_	0.67		0.67	
*R*^2^_0_	0.15		0.14	
*R*^2^_1_	0.20		0.13	
*R*^2^_2_	0.25		0.00	

∧ p < 0.10;

* p < 0.05;

** p ≤ 0.01;

*** p < 0.001.

To further specify the nature of the interaction between concentrated disadvantage and gender, Model A was run twice with gender re-centered, one model with males and females coded as 0 and 1, respectively, and one with males and females were coded as 1 and 0. Consequently, the slope coefficient of concentrated disadvantage in each model is for that gender coded as 0. With male coded as 0, and coefficients for disadvantage thus reflecting the effect for males, higher concentrated disadvantage predicted increased cortisol reactivity (*B* = 0.006, *p* = 0.004, *r*_effect_ = 0.33) and a steeper decline during recovery (*B* = −0.003, *p* = 0.003, *r*_effect_ = 0.34). No effects were observed for the intercept (*B* = −0.06, *p* = 0.43, *r*_effect_ = 0.09). With females coded as 0, and coefficients for disadvantage thus reflecting the effect for females, the effect of concentrated disadvantage was not significant for the intercept (*B* = −0.09, *p* = 0.29, *r*_effect_ = 0.13), reactivity (*B* = 0.0004, *p* = 0.85, *r*_effect_ = 0.02) or recovery (*B* = 0.001, *p* = 0.40, *r*_effect_ = 0.10).

**Figure 2:**
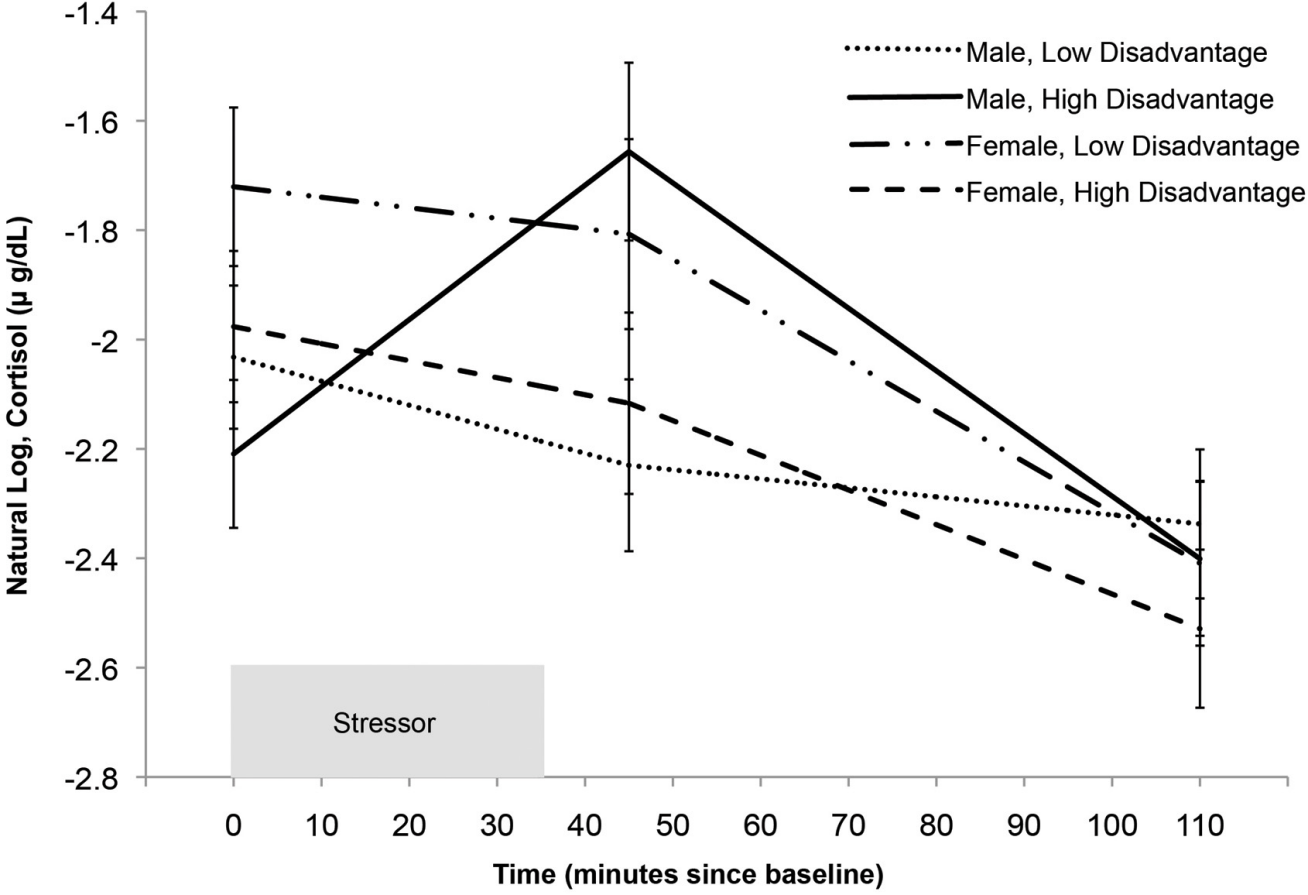
**Predicted cortisol reactivity by gender and level of concentrated disadvantage.** Model-based graphs of cortisol concentration during the reactivity and recovery periods protocol by gender and level of concentrated disadvantage. This model controls for the effects of sleep and asthma medication (non-steroidal). For illustrative purposes we selected two values representing high and low disadvantage, 1.5 SD above and below the mean (following Aiken and West, 1991). Error bars represent standard error.

Following Aiken and West (1991), we selected values of concentrated disadvantage that are 1.5 SD's above and below the grand mean to illustrate the interaction effect in Figure 2. Notably, the intraclass correlation coefficient (ICC) for cortisol in males was 0.65, while the ICC for females was 0.52. This is indicative of considerable between-subject variability in both genders, suggesting the interaction is not explained by the absence of variability between subjects in females, but nevertheless more variability in cortisol is explained by between subjects factors in males than in females. In addition, it is unlikely that these results are explained by maturational differences, as age is neither correlated with disadvantage (*r* = 0.11, *p* = 0.37) nor gender (*t* = −0.46, *p* = 0.64).

### Independence from subjective response

Do the differences observed in cortisol response, with high reactivity among low SES boys, reflect differences in physiological response to subjectively appraised stressors of a given intensity, or differences in the subjective appraisals themselves? A series of models was run to determine if any such factors explained the observed interaction in Model A. Self-rated anxiety at Level 1, measured concurrently with salivary cortisol and added to Model A, did not predict salivary cortisol (*B* = −0.008, *p* = 0.33, *r*_effect_ = 0.04). Next, a set of models were run in which measures of subjective appraisal and response to the stressor as well their interaction with gender were added to Model A at Level 2. In these models neither anxiety reactivity (peak minus baseline) (*B* = −0.00006, *p* = 0.94, *r*_effect_ = 0.01), the appraisal of the stressor (*B* = −0.0002, *p* = 0.78, *r*_effect_ = 0.03), math difficulty ratings (*B* = −0.0004, *p* = 0.61, *r*_effect_ = 0.06), or speech difficulty ratings (*B* = 0.001, *p* = 0.07, *r*_effect_ = 0.22) predicted reactivity. In addition, none of the interactions between these indices of subjective appraisal and gender were significant predictors of reactivity (all *p* > 0.27). With respect to recovery, neither anxiety reactivity (*B* = 0.0001, *p* = 0.80, *r*_effect_ = 0.03), the appraisal of the stressor (*B* = −0.00005, *p* = 0.91, *r*_effect_ = 0.01), math difficulty ratings (*B* = 0.0003, *p* = 0.50, *r*_effect_ = 0.08), or speech difficulty ratings (*B*= −0.00004, *p* = 0.94, *r*_effect_ = 0.01) were significant predictors. As with reactivity, none of the interactions between these indices of subjective appraisal and gender were significant predictors of recovery reactivity (all *p* > 0.19). In all models the interaction between gender and disadvantage remained a significant predictor of reactivity (all *p* ≤ 0.047) and recovery (all *p* ≤ 0.009).

## Discussion

In this analysis we found that concentrated neighborhood disadvantage, but not parental education, was associated with cortisol reactivity and recovery. However, this relationship was moderated by gender, such that higher concentrated disadvantage was associated with higher cortisol reactivity and steeper recovery in boys alone. This association was not explained by differences in subjective reactivity to the stressor. This highlights the particular importance of neighborhood effects and that SES differences in brain development and particularly reactivity to environmental stressors may vary across genders.

The direction of this association is consistent with prior studies in children and adolescents indicating that lower SES, particularly low income, predicts greater stress reactivity (Blair et al., 2005; Gump et al., 2009). In addition, it may help to reconcile prior reports with the null findings on stress reactivity of a quasi-experimental intervention composed of cash payments as well as health and educational programs (Fernald and Gunnar, 2009). In particular, analyses of income effects that do not measure and include neighborhood disadvantage may find income effects that are a proxy for neighborhood effects, given their correlation; if this were the case, an intervention targeting income would be expected to produce null results. However, it remains possible that this intervention did not predict reactivity because it was a more rigorous design that better accounted for additional unobserved variables, and a direct test of the relative associations of income and neighborhood disadvantage remains to completed. Somewhat surprising was the absence of a relationship between SES indicators and baseline cortisol prior to the stressor, though this is likely explained by the fact that most SES effects are found on broader measures of diurnal function and that null results have been found previously in adolescents (Lupien et al., 2000, 2001; Evans and English, 2002; Chen and Paterson, 2006; Gustafsson et al., 2006; Evans and Kim, 2007; Zalewski et al., 2012).

The positive relationship between neighborhood disadvantage and stress reactivity is consistent with the Biological Sensitivity to Context and Adaptive Calibration models of stress reactivity (Del Giudice et al., 2011; Ellis and Boyce, 2008), which predict higher levels of responsivity in more stressful, dangerous environments, as well as possible gender differences which emerge across development. These models may also help integrate these findings within the broader adversity literature, particularly that concerning abuse, which has often been found to be predict decreased cortisol reactivity (Carpenter et al., 2007, 2009; Elzinga et al., 2008), though this is not always the case (Heim et al., 2000). In particular, the adaptive calibration model distinguishes between the stressful, unpredictable conditions associated with lower SES and neighborhood disadvantage, which are predicted to promote increased reactivity to stress, consistent with the current findings and in contrast with more severe or traumatic stress that promotes an unresponsive pattern (Del Giudice et al., 2011).

In addition, increased reactivity in boys from neighborhoods with high levels of disadvantage may increase their vulnerability to environmental effects (Ellis and Boyce, 2008; Belsky and Pluess, 2009), thereby increasing the likelihood of future problems given the stressful and unpredictable neighborhoods they are exposed to. Moreover, in a meta-analysis, Chida and Hamer (2008) found that reduced HPA-axis reactivity was a predictor of positive psychological traits and states. However, studies have found that increased reactivity is predictive of better executive function and mood (Blair et al., 2005; Burke et al., 2005). Much remains to be understood about the role of stress reactivity in important life outcomes, but it seems likely that the functional importance of such increased reactivity may depend on the outcome domain and the future environments adolescents are exposed to.

The specificity of the association between neighborhood disadvantage and reactivity suggests that the family resources associated with education do not underlie differences in reactivity. It remains to be determined what mechanism is driving these effects in boys. It has been suggested that boys are more vulnerable to neighborhood disadvantage due to differences in the amount of unsupervised free time allowed by parents (Hilbrecht et al., 2008; Leventhal and Brooks-Gunn, 2011). Peer effects are another candidate mediator, as social status among peers, moderated by gender, is a better predictor of morning cortisol than family SES (West et al., 2010). In addition, any potentially causal effects of neighborhood disadvantage may be mediated by parenting practices (Repetti et al., 2002; Luecken and Lemery, 2004; Hackman et al., 2010) or the manner in which stressors are cognitively framed (Chen et al., 2012). Through any such mechanism, it is likely that effects are transmitted at least in part through changes in gene expression for the glucocorticoid receptor leading to heightened responses to stress (Miller et al., 2009).

One potential limitation to interpretation of the interaction between gender and neighborhood disadvantage is the association of gender and reactivity, in which adolescent females exhibited smaller responses to the stressor overall. This is consistent with prior findings that females exhibit smaller responses to such performance based stress protocols, rather than social rejection-based protocols (Kirschbaum et al., 1999; Stroud et al., 2002; Dedovic et al., 2009). As such, it could be argued that the interaction is primarily due to the lack of response among females and that only a main effect of disadvantage would be observed with a different stressor protocol. However, despite the overall main effect of gender, the intraclass correlation within females indicates that nearly half of the variability of cortisol across time is between subjects, suggesting considerable variability exists to predict systematic differences. Concentrated disadvantage, however, does not significantly predict such differences in females. Moreover, moderation of neighborhood effects by gender is consistent with quasi-experimental and observational studies of neighborhood effects on children and adolescents (Kling et al., 2007; Leventhal and Brooks-Gunn, 2011) as well as experimental literature in animals indicating that the impact of environmental stressors on the HPA axis is moderated by gender (McCormick and Mathews, 2007; Weinstock, 2007; Lin et al., 2009). Nevertheless, the stability of this interaction across stressor types bears empirical investigation.

As with all observational studies on SES, it is impossible to firmly establish the direction of causality, as similar heritable factors may influence both socioeconomic position and stress reactivity. However, multiple lines of evidence suggest this effect is likely to be due to social causation, at least in part. First, animal literature experimentally demonstrates the effect of environment on stress reactivity (Zhang et al., 2006). In addition, twin studies suggest that environmental factors are the primary determinants of the stress response during the first exposure to a stressor, especially for those raised under conditions of adversity (Federenko et al., 2004; Ouellet-Morin et al., 2008; Steptoe et al., 2009).

Despite the specificity of the relationships demonstrated here between neighborhood disadvantage and cortisol reactivity and recovery, we recognize that such claims warrant caution. In particular, although we employed a measure of neighborhood concentrated disadvantage based on the child's census tract of residence, the number of participants in each tract was too small to treat individuals as nested within the neighborhood as a higher level of organization (Subramanian et al., 2003). Consequently, this analysis is not able to model the inherently multilevel nature of neighborhood effects.

In summary, SES as indexed by concentrated neighborhood disadvantage is associated with cortisol reactivity and recovery in boys, but not in girls. These findings suggest that the mechanisms underlying SES differences in the neural systems underlying stress regulation vary across genders.

### Conflict of interest statement

The authors declare that the research was conducted in the absence of any commercial or financial relationships that could be construed as a potential conflict of interest.

## Footnotes

^1^Subsample analyses found that a piecewise model was a better fit for the data than a quadratic model of change (data not shown).

^2^Subsample analyses found similar effects employing alternative analysis approaches, including a mixed model ANOVA and area under the curve as compared to baseline, a summary measure of reactivity (data not shown).
